# Temporal trend and factors of C-section and its impact on subsequent pregnancy outcomes in Central China

**DOI:** 10.3389/fmed.2025.1683485

**Published:** 2025-12-11

**Authors:** Hui Li, Shi Yuanmei, Xiaoli Tang, Hong Zhang, Nawsherwan, Lu Xinhua

**Affiliations:** 1 Department of Medical, Taixing People's Hospital Affiliated to Yangzhou University, Taizhou, Jiangsu, China; 2 Department of Paediatrics, Taixing People's Hospital, Taizhou, Jiangsu, China; 3 Department of Administration, Taixing People's Hospital, Taizhou, Jiangsu, China; 4 Department of Neonatology, Taixing People's Hospital, Taizhou, Jiangsu, China; 5 Xiamen Cardiovascular Hospital of Xiamen University, School of Medicine, Fujian Branch of National Clinical Research Centre for Cardiovascular Diseases, Xiamen, China

**Keywords:** C-section, secular trend, risk factors, subsequent pregnancy outcomes, singleton, twins

## Abstract

**Background:**

Cesarean section (C-section) rates have increased dramatically over the last few decades worldwide, and China's C-section rate has continuously remained much higher than the World Health Organization (WHO)'s suggested threshold. This study aimed to (1) estimate the temporal trends and factors for C-sections and (2) examine the association of a previous C-section with adverse outcomes in a subsequent pregnancy.

**Methods:**

We conducted a tertiary-level hospital-based retrospective cohort study in the Hubei Province, China between 2011 and 2019. A chi-squared test was used to estimate the baseline disparities between C-sections and normal delivery among singleton and twin pregnancies. A multiple binary logistic regression model was conducted to predict factors associated with C-sections and their impact on subsequent pregnancy outcomes. The secular trend of C-section was determined by using the joinpoint regression model.

**Results:**

Between 2013 and 2019, the trend of C-section was significantly increased among singleton women with a high education level (annual percentage change (APC), 9.8%; 95%CI: 5.3, 14.5) and professional services (APC, 7.9%; 95%CI: 2.9, 13.2). The leading five factors, including previous C-section followed by fetal breech presentation, abnormal placentation, oligohydramnios, and macrosomia, were associated with a higher risk of C-section among singleton pregnancies. Hypertensive disorders of pregnancy (HDP) increase the risk of C-section in women with twin gestations. Previous C-section associated with a higher likelihood of gestational diabetes mellitus (GDM) in the subsequent pregnancy of singleton (aOR, 1.30; 95%CI: 1.14, 1.49) and twin (aOR, 1.78; 95%CI: 1.05, 3.01) women. Moreover, previous C-sections increased the odds of C-sections (aOR, 3.41; 95%CI: 3.11, 3.75) in the subsequent pregnancy of singleton women.

**Conclusion:**

The secular trend of C-sections significantly increased among women with high socioeconomic status, and a previous C-section was associated with a higher likelihood of repeated C-sections in singleton women and GMD in the subsequent pregnancy of both singleton and twin women.

## Introduction

Cesarean section (C-section), as a mode of delivery, has significantly increased over the last few decades worldwide (1). In the last two and a half decades (1990–2014), 18.6% of all neonatal births occurred by C-section across 150 countries, including the least developed (i.e., showing C-section rate by 6.0%) and most developed regions (i.e., showing C-section rate by 27.2%). The absolute increase in the C-section rate occurred by 12.4%, from 6.7% in 1990 to 19.1% in 2014 globally. Latin America and the Caribbean region observed the most significant absolute increase in the C-section rate (19.4%), followed by Asia (15.1%), Oceania (14.1%), Europe (13.8%), North America (10.0%), and Africa (4.5%). Moreover, Asia showed the highest average annual rate of increase in the C-section rate (6.4%) among the aforementioned world regions (1).

China had the highest rate of C-section (46.2%) in a World Health Organization (WHO) survey during 2007–2008 (2), which was threefold higher than the WHO's target of 15.0% (3) and showed 11.6% non-medically indicated C-section (2). Between 1988 and 2008, the C-section rate increased from 3.4% to 39.3% at the national level, with the highest C-section rate in urban areas (64.1%) than in rural areas (11.3%) in 2008 (4). Moreover, the C-section rate increased from 2.0% in 1978 to 54.9% in 2011 (5, 6). In a multicenter retrospective cohort study, Song et al. (7) reported that the C-section rate declined from 52.5% in 2012–2015 to 49.7% in 2016–2019. Although there have been fluctuations in the C-section rate over the last few decades, China's C-section rate has consistently remained far higher than the recommended limit established by the WHO (3).

Several factors are associated with an increased risk of C-section, including maternal factors (i.e., anxiety about labor, fear of delivery pain, advanced maternal age (AMA), history of previous C-section, obesity, gestational diabetes mellitus (GDM), gestational hypertension, preeclampsia, eclampsia, placenta previa, placenta accrete, placental abruption, etc.), and neonatal factors (i.e., macrosomia, breech or abnormal fetal presentation, and non-reassuring fetal heart rate) (8, 9). Previous studies suggest that babies born by C-section showed adverse health outcomes in later life, including type-1 diabetes mellitus (10), respiratory morbidity (11), asthma (12), allergies (13), and obesity (6). Furthermore, a previous C-section is significantly associated with an increased risk of gestational hypertension, preeclampsia, polyhydramnios (14), placenta previa, labor dystocia, intrapartum hemorrhage, primary postpartum hemorrhage, and C-section in the subsequent pregnancy (15).

Several studies have been published on C-sections in China (5–7, 9, 16–19); however, limited studies (20–24) have estimated the impact of C-sections on the adverse maternal–perinatal outcomes in the subsequent pregnancy. Therefore, this study aimed to estimate the secular trend and factors of C-sections and the impact of previous C-sections on the subsequent pregnancy outcomes among women with singleton and twin gestations in Hubei, China, between 2011 and 2019.

## Materials and methods

### Study design and participants

The present retrospective cohort study was conducted in a tertiary-level hospital, Department of Obstetrics and Gynecology, Hubei, China, according to the strengthening of the reporting of observational studies in epidemiology (STROBE) guidelines between 2011 and 2019 (25). Trained and experienced nurses gathered and recorded the data on pregnant women (*n* = 25,678) in the obstetrics register and electronic database while they were being examined in the Gynecology and Obstetrics Department. The study protocol was approved by the Ethical Review Board of tertiary-level hospital in the Hubei province (ID: WDRY2019–034) in accordance with the Declaration of Helsinki.

### Inclusion and exclusion criteria

**Figure 1:**
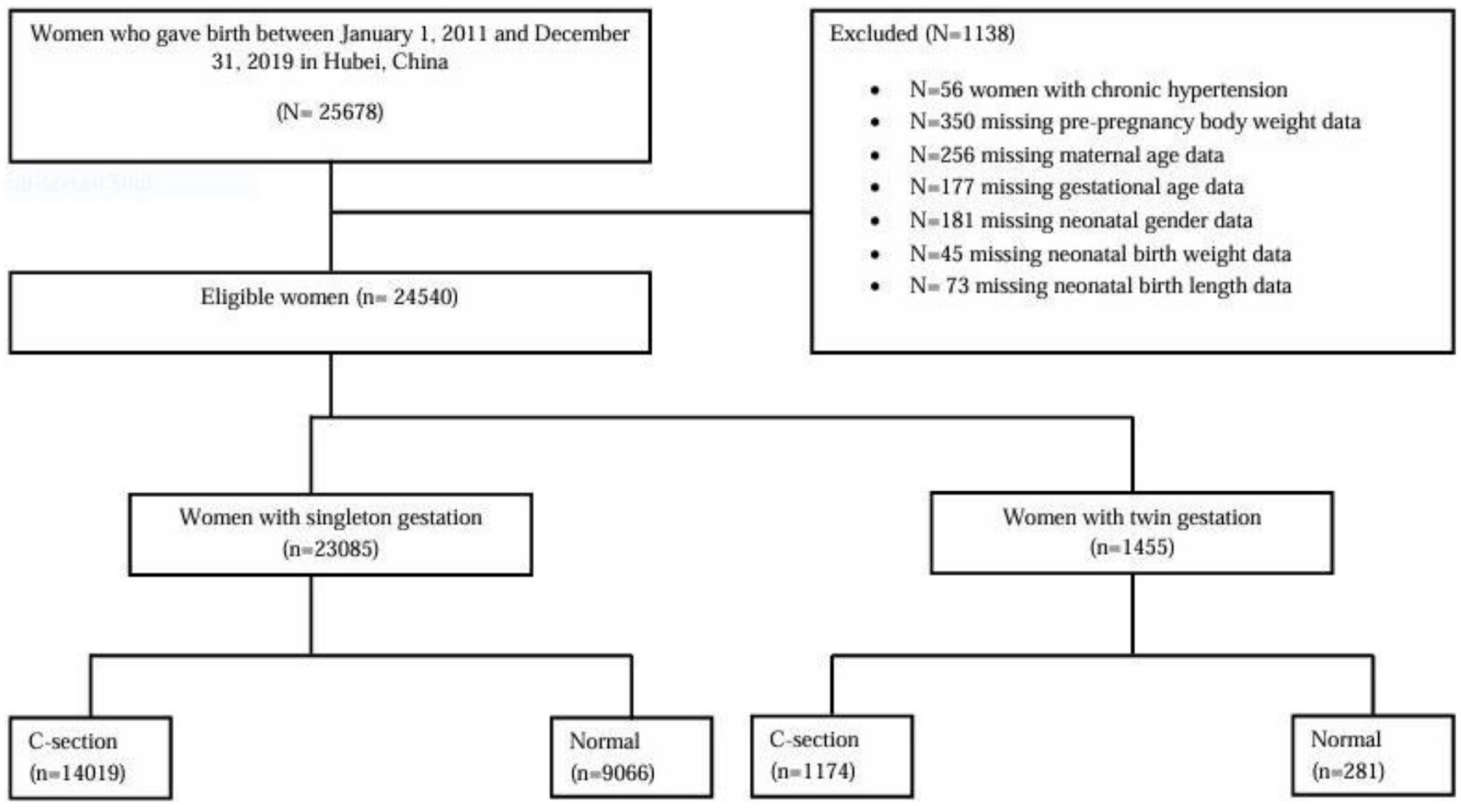
Flowchart of the study population.

A total of 24,540 pregnant women, including women with singleton gestation (*n* = 23,085) and twin gestations (*n* = 1,455), were selected for the current study. The analysis included both live births and neonatal deaths, encompassing full-term and preterm (< 37 weeks) neonates, as well as those with low birth weight (< 2,500 g). The data pertaining to chronic hypertension, as well as any missing information on maternal age, gestational age, pre-pregnancy body weight, and neonatal-related variables such as gender, birth weight, and length at delivery, were excluded from the statistical analysis, as illustrated in Figure 1. In order to solve the missing data in our study, which was missing at random (MAR), we used the listwise or case deletion technique, which entails eliminating subjects with incomplete data and analyzing the remaining data (26).

### Data collection

The data were collected from the obstetrics register and electronic database regarding maternal–neonatal characteristics and outcomes, including maternal age, education, occupation, parity, pre-pregnancy body weight, gestational age, neonatal gender, birth weight, birth length, and pregnancy outcomes including maternal outcomes (i.e., C-section, previous history of C-section, GDM, HDP, abnormal placentation, premature rupture of membrane (PROM), nuchal cord, oligohydramnios, polyhydramnios, fetal breech presentation), and neonatal outcomes (i.e., preterm birth, low birth weight [LBW], low ponderal index [LPI], perinatal mortality, macrosomia, low Apgar score, fetal distress, intrauterine growth restriction [IUGR], and congenital defects). Maternal education is divided into three categories based on the length of formal education completed: low ( ≤ 8 years), middle (9–12 years), and higher (≥13 years). Maternal occupations were categorized as (i) housewives, (ii) professional services (i.e., composite of doctors, nurses, accountants, teachers, lawyers, and actresses), and (iii) manual workers (i.e., composite of farmers, waitresses, drivers, and factory workers) (27). The detailed definitions of maternal–perinatal outcomes have been described in our previous articles (28–30) and Supplementary material. Based on prior research, confounding variables such as maternal age, pre-pregnancy body weight ( ≤ 45 kg and >91 kg), parity, and neonatal gender were selected for this analysis (31).

### Statistical analysis

In the first step, we conducted a chi-squared test to find the disparities in the general characteristics and maternal–neonatal outcomes by mode of delivery (i.e., normal delivery and C-section) among singleton and twin pregnancies. For categorical and binary variables, descriptive statistics such as frequencies and percentages were computed. In the second step, multiple binary logistic regression models were used (adjusted for parity, education, occupation, pre-pregnancy body weight, and neonatal gender) to determine the factors associated with C-sections among women with singleton and twin gestations. In this model, C-section was an outcome variable. The predictor variables were maternal–perinatal outcomes, including advanced maternal age (AMA), GDM, HDP, abnormal placentation, previous history of C-section, PROM, nuchal cord, oligohydramnios, polyhydramnios, fetal breech presentation, macrosomia, fetal distress, IUGR, and congenital defects. In the third step, we restricted the analysis to multiparous women and employed multiple binary logistic regression models to assess the association between a previous C-section and subsequent pregnancy outcomes, adjusting for maternal age, education, occupation, pre-pregnancy body weight, and neonatal gender. In this model, the previous C-section was a predictor variable, and the outcome variables were maternal–neonatal outcomes, as mentioned earlier. The associations between predictor and outcome variables were estimated using the adjusted odds ratios (aOR) with 95% confidence intervals. *P*-value (two-tailed < 0.05) was considered statistically significant. The data were analyzed by using SPSS (Statistical Package for Social Sciences) for Windows version 22 (IBM Corporation, New York, USA).

In the fourth step, we used joinpoint regression analysis to find the secular trend of C-sections among women with singleton and twin gestations between 2011 and 2019. Moreover, we determined the secular trend of C-sections based on education levels and professional status among singleton and twin pregnancies. The percentage changes (APC) and average annual percentage changes (AAPC) were estimated for C-section in the joinpoint regression analysis. The APC shows the trend in the C-section in each segment, and the AAPC indicates a trend in the C-section in the whole study period, 2011–2019. The APPC or APC >0 with its 95% confidence interval (CI) shows a positive secular trend; however, the APPC or APC < 0 with its 95% CI shows a negative secular trend. A joinpoint regression model with one joinpoint was selected as the optimal model, as it yielded the lowest values for both the Bayesian Information Criterion (BIC) and Akaike Information Criterion (AIC). Moreover, Monte Carlo methods were used to find each *p*-value and maintain the overall asymptotic significance level through Bonferroni correction. The joinpoint regression analysis was conducted using the joinpoint regression program version 4.8.0.1 (April 2020) from the Surveillance Research Program of the U.S. National Cancer Institute.

## Results

### Distribution of general characteristics and pregnancy outcomes by mode of delivery among singleton and twin pregnancies

A total of 24,540 pregnant women were selected, including singleton (*n* = 23,085) and twin (*n* = 1,455) gestations. Among the singletons, 60.7% of women had a C-section. Women with C-sections were significantly older (i.e., AMA) (20.8% vs. 11.4%) and showed a higher incidence of GDM (7.8% vs. 4.9%), HDP (8.0% vs. 4.1%), abnormal placentation (6.0% vs. 1.9%), oligohydramnios (4.3% vs. 2.2%), fetal breach presentation (3.4% vs. 1.1%), macrosomia (6.8% vs. 3.3%), and fetal distress (2.7% vs. 1.6%) compared with normal women. However, women with C-sections had significantly lower incidence of preterm birth, LBW, perinatal mortality, LPI, and low Apgar scores compared with normal women. Among twin gestations, 80.7% of women experienced C-sections. Women with C-sections had a higher incidence of HDP (13.7% vs. 3.9%) and showed a lower incidence of preterm birth, LBW, perinatal mortality, LPI, and low Apgar score compared with normal women (Tables 1, 2).

**Table 1:** Baseline maternal characteristics and pregnancy outcomes among singleton and twin pregnancies by mode of delivery (*N* = 24,540).

**Maternal characteristics and pregnancy outcomes**	**Singleton (*****n*** = **23,085)**			**Twins (*****n*** = **1,455)**		
	**Normal (*****n*** = **9,066)** ***n*** **(%)**	**C-section (*****n*** = **14,019)** ***n*** **(%)**	***P*** **-value**	**Normal (*****n*** = **281)** ***n*** **(%)**	**C-section (*****n*** = **1174)** ***n*** **(%)**	***P*** **-value**
Maternal age			< 0.001			0.1
< 35 years	8,032 (88.6)	11,097 (79.2)		238 (84.7)	952 (81.1)	
≥35 years	1,034 (11.4)	2,922 (20.8)		43 (15.3)	222 (18.9)	
Parity			< 0.001			0.4
Primiparous ( ≤ 1)	7,172 (79.1)	10,342 (73.8)		221 (78.6)	950 (80.9)	
Multiparous (>1)	1,894 (20.9)	3,677 (26.2)		60 (21.4)	224 (19.1)	
Maternal education			0.003			0.1
Higher	3,660 (40.4)	5,386 (38.4)		83 (29.5)	426 (36.3)	
Middle	3,543 (39.1)	5,543 (39.5)		118 (42.0)	439 (37.4)	
Low	1,863 (20.5)	3,090 (22.1)		80 (28.5)	309 (26.3)	
Maternal occupation			< 0.001			0.7
Professional services	4,255 (46.9)	6,217 (44.3)		113 (40.2)	490 (41.7)	
Manual workers	224 (2.5)	324 (2.3)		5 (1.8)	28 (2.4)	
House wives	4,587 (50.6)	7,478 (53.4)		163 (58.0)	656 (55.9)	
GDM^*^	448 (4.9)	1,090 (7.8)	< 0.001	27 (9.6)	107 (9.1)	0.8
HDP^*^	372 (4.1)	1,128 (8.0)	< 0.001	11 (3.9)	161 (13.7)	< 0.001
Abnormal placentation^*^	173 (1.9)	844 (6.0)	< 0.001	8 (2.8)	31 (2.6)	0.8
Previous history of C-section^*^	620 (6.8)	2,975 (21.2)	< 0.001	23 (8.2)	132 (11.3)	0.1
PROM^*^	1,044 (11.5)	1,109 (7.9)	< 0.001	56 (20.0)	114 (9.7)	< 0.001
Nuchal cord^*^	564 (6.2)	432 (3.1)	< 0.001	15 (5.3)	62 (5.3)	1.0
Oligohydramnios^*^	196 (2.2)	609 (4.3)	< 0.001	2 (0.7)	7 (0.6)	0.6
Polyhydramnios^*^	24 (0.3)	67 (0.5)	0.01	3 (1.1)	6 (0.5)	0.3
Fetal breech presentation^*^	98 (1.1)	477 (3.4)	< 0.001	6 (2.1)	34 (2.9)	0.6

*p*-values were calculated using the chi-squared test. ^*^Frequency and percentage of variables with only “yes” value presented: maternal age ≥35 years, advanced maternal age; HDP, hypertensive disorders of pregnancy; GDM, gestational diabetes mellitus; PROM, premature rupture of membrane.

**Table 2:** Baseline perinatal outcomes among singleton and twin pregnancies by mode of delivery.

**Perinatal outcomes**	**Singleton (*****n*** = **23,085)**			**Twins (*****n*** = **1,455)**		
	**Normal (*****n*** = **9,066)** ***n*** **(%)**	**C-section (*****n*** = **14,019)** ***n*** **(%)**	***P*** **-value**	**Normal (*****n*** = **281)** ***n*** **(%)**	**C-section (*****n*** = **1174)** ***n*** **(%)**	***P*** **-value**
Preterm birth^*^	1,659 (18.3)	2,771 (19.8)	0.006	252 (89.7)	807 (68.7)	< 0.001
LBW^*^	1,387 (15.3)	1,896 (13.5)	< 0.001	242 (86.1)	701 (59.7)	< 0.001
Perinatal mortality^*^	187 (2.1)	146 (1.0)	< 0.001	12 (4.3)	21 (1.8)	0.02
LPI^*^	418 (4.6)	480 (3.4)	< 0.001	60 (21.4)	71 (6.0)	< 0.001
Macrosomia^*^	298 (3.3)	954 (6.8)	< 0.001	2 (0.7)	0 (0)	0.03
Low Apgar score^*^	485 (5.3)	359 (2.6)	< 0.001	95 (33.8)	134 (11.4)	< 0.001
Fetal distress^*^	144 (1.6)	378 (2.7)	< 0.001	1 (0.4)	17 (1.4)	0.2
IUGR^*^	62 (0.7)	106 (0.8)	0.5	1 (0.4)	5 (0.4)	1.0
Congenital defects^*^	143 (1.6)	155 (1.1)	0.002	2 (0.7)	6 (0.5)	0.6
Neonatal gender			0.1			0.2
Male	4,795 (52.9)	7,545 (53.8)		138 (49.1)	626 (53.3)	
Female	4,271 (47.1)	6,474 (46.2)		143 (50.9)	548 (46.7)	

*p*-values were calculated using the chi-squared test. ^*^Frequency and percentage of variables with only “yes” value presented: LBW, low birth weight; IUGR, intrauterine growth restriction; LPI, low ponderal index; ^*^congenital defects [microtia, anotia, polydactyly, heart defects, limb reduction defects, cleft lip, cleft palate, hydrocephaly, and neural tube defects (NTDs)].

### Factors for C-sections among singleton and twin pregnancies

**Figure 2:**
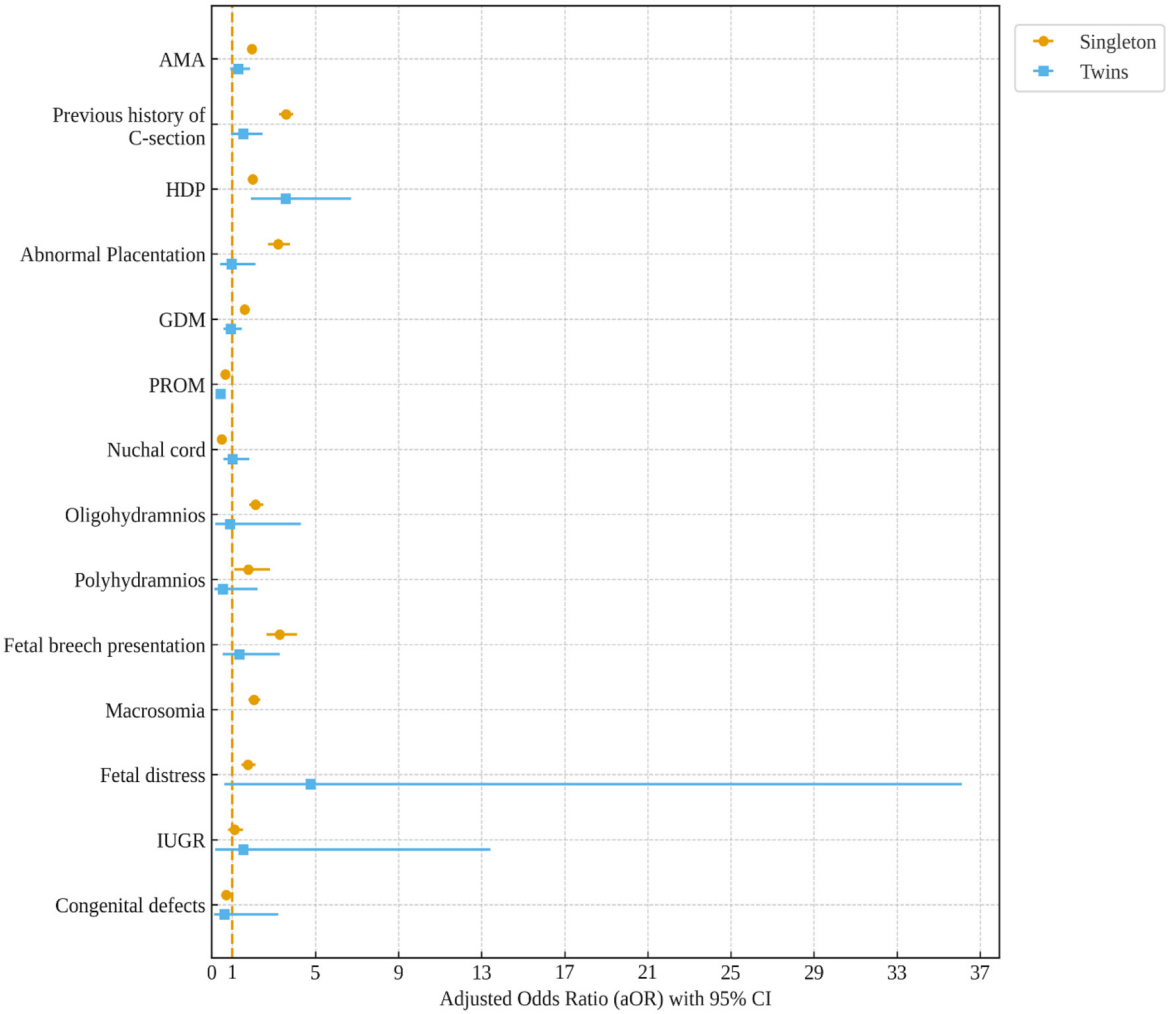
Factors for C-section among singleton and twin pregnancies. Models were adjusted for multiparity, education, occupation, pre-pregnancy body weight, and neonatal gender. AMA, advanced maternal age; HDP, hypertensive disorders of pregnancy; GDM, gestational diabetes mellitus; PROM, premature rupture of membranes; IUGR, intrauterine growth restriction.

Among singleton pregnancies, AMA (aOR, 1.94; 95%CI: 1.80, 2.10), previous history of C-section (aOR, 3.59; 95%CI: 3.27, 3.94), HDP (aOR, 1.98; 95%CI: 1.75, 2.24), abnormal placentation (aOR, 3.21; 95%CI: 2.71, 3.79), GDM (aOR, 1.59; 95%CI: 1.42, 1.78) oligohydramnios (aOR, 2.12; 95%CI: 1.80, 2.50), polyhydramnios (aOR, 1.76; 95%CI: 1.10, 2.82), fetal breech presentation (aOR, 3.29; 95%CI: 2.64, 4.11), macrosomia (aOR, 2.04; 95%CI: 1.79, 2.34), and fetal distress (aOR, 1.75; 95%CI: 1.44, 2.12) were associated with higher odds of C-section. Moreover, HDP (aOR, 3.58; 95%CI: 1.91, 6.72) significantly increased the risk of C-section among women with twin gestations (Figure 2).

### Association of previous C-sections with subsequent adverse pregnancy outcomes among singleton and twin pregnancies

**Figure 3:**
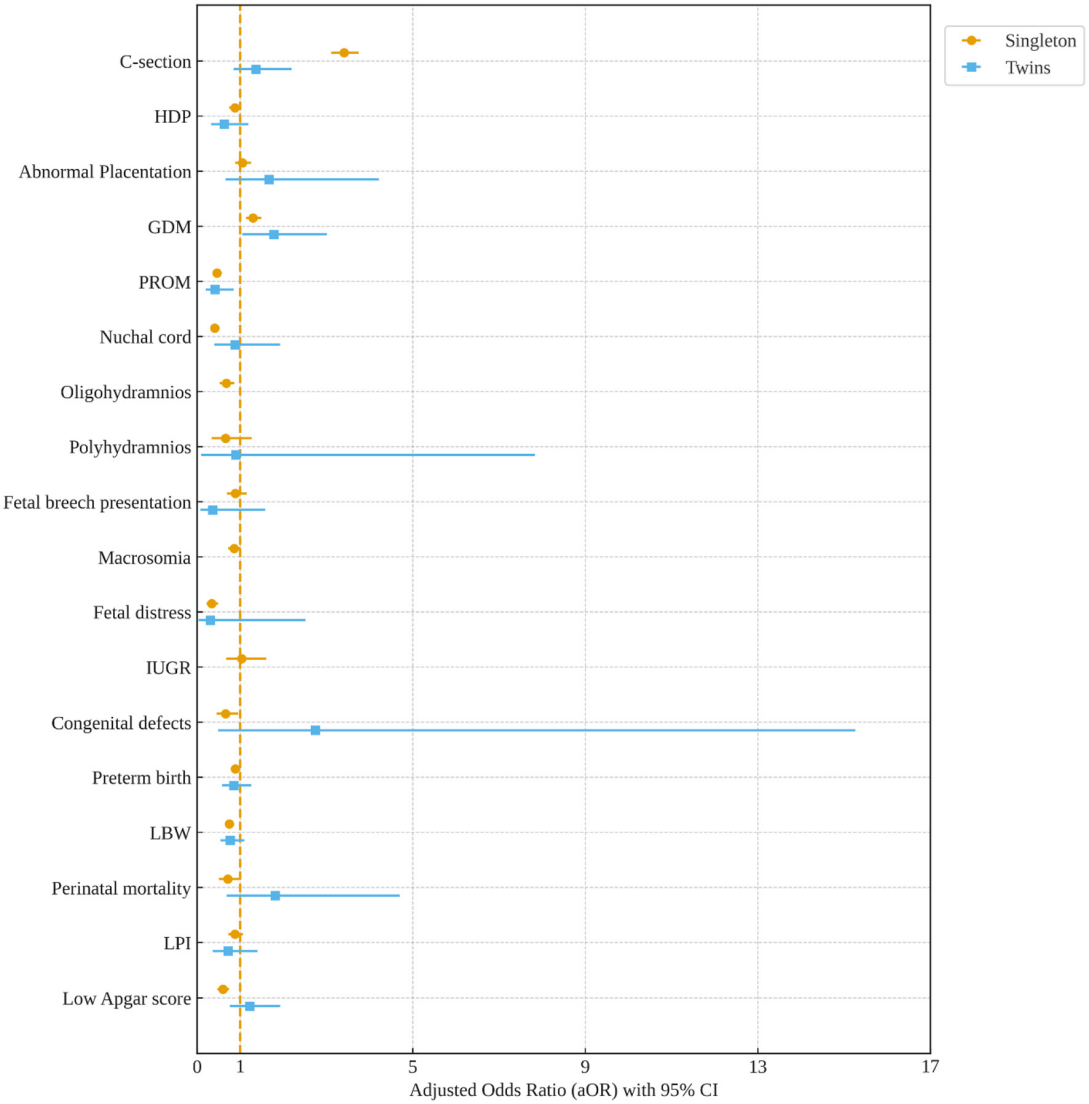
Association of previous C-section with subsequent adverse pregnancy outcomes among singleton and twin pregnancies. Models were adjusted for AMA, education, occupation, and pre-pregnancy body weight. HDP, hypertensive disorders of pregnancy; GDM, gestational diabetes mellitus; PROM, premature rupture of membrane; LBW, low birth weight; IUGR, intrauterine growth restriction; LPI, low ponderal index.

Women with a previous history of C-section significantly increased the odds of GDM among singleton (aOR, 1.30; 95%CI: 1.14, 1.49) and twin (aOR, 1.78; 95%CI: 1.05, 3.01) pregnancies. Moreover, previous C-sections increased the odds of C-sections (aOR, 3.41; 95%CI: 3.11, 3.75) in the following pregnancy among women with singleton gestation. However, a previous history of C-sections is associated with a lower odds of PROM, nuchal cord, preterm birth, LBW, perinatal mortality, and low Apgar score among singleton pregnancies (Figure 3).

### Secular trend of C-section among singleton and twin pregnancies (2011–2019)

**Figure 4:**
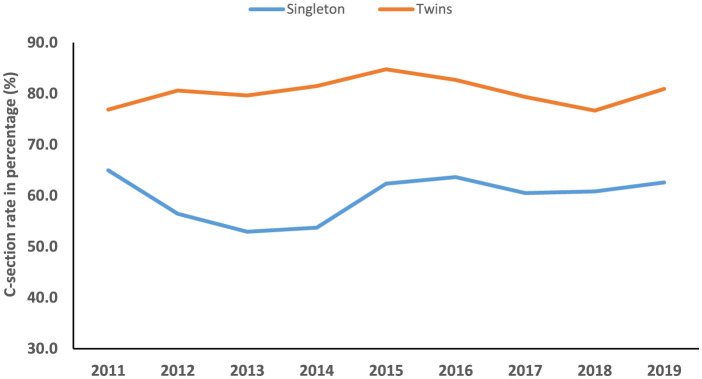
Temporal trend of C-section among singleton and twin pregnancies (2011–2019).

**Figure 5:**
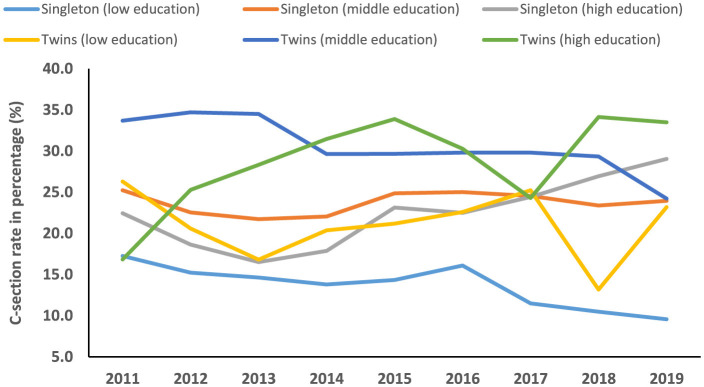
Temporal trend of C-section among singleton and twin pregnancies with different education levels (2011–2019).

**Figure 6:**
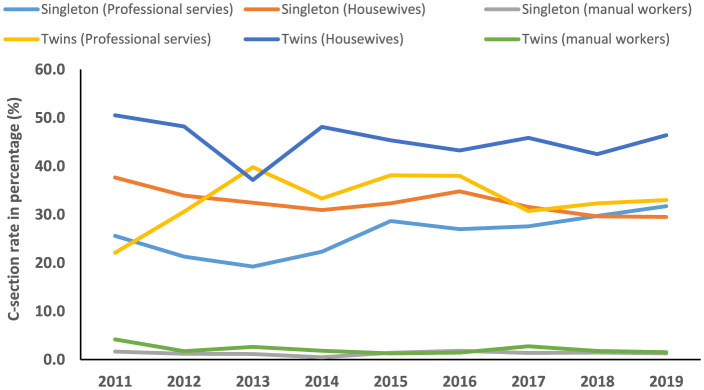
Temporal trend of C-section among singleton and twin pregnancies with different occupation status (2011–2019).

The temporal trend of C-section among singleton (average annual percent change (AAPC), 0.2%; 95%CI: −4.2, 4.7) and twin pregnancies (AAPC, 0.1%; 95%CI: −1.6, 1.9) was not significantly increased between 2011 and 2019. Among twins with middle education levels, the trend of C-sections was significantly decreased (AAPC, −3.8%; 95%CI: −7.4, −0.1) during the study period. However, the trend of C-sections was significantly increased among singleton women with a high education level (annual percentage change (APC), 9.8%; 95%CI: 5.3, 14.5) and professional services (APC, 7.9%; 95%CI: 2.9, 13.2) between 2013 and 2019 (Tables 3, 4, Figures 4–6).

**Table 3:** Secular trend of C-section among singleton and twin pregnancies from 2011 to 2019.

**Population and variables**	**Year**	**APC (95% CI)**
**Singleton**		
Trend1	2011–2013	−7.5 (−26.0, 15.5)
Trend2	2013–2019	2.8 (−0.9, 6.8)
AAPC (95%CI)	2011–2019	0.2 (−4.2, 4.7)
**Twins**		
Trend1	2011–2015	1.8 (−1.7, 5.5)
Trend2	2015–2019	−1.6 (−5.0, 1.9)
AAPC (95%CI)	2011–2019	0.1 (−1.6, 1.9)

APC, annual percentage change; AAPC, average annual percent change; CI, confidence interval.

**Table 4:** Secular trend of C-section by education levels and occupation status among singleton and twin pregnancies from 2011 to 2019.

**Education**	**Singleton**		**Twins**	
	**Year**	**APC (95% CI)**	**Year**	**APC (95% CI)**
**Low**				
Trend1	2011–2016	−2.4 (−6.0, 1.4)	2011–2013	−13.4 (−18.1, 35.1)
Trend2	2016–2019	−13.3 (−20.4, −5.6)^*^	2013–2019	1.3 (−14.4, 20.0)
AAPC (95%CI)	2011–2019	−6.6 (−9.2, −3.9)^*^	2011–2019	−2.6 (−20.0, 18.7)
**Middle**				
Trend1	2011–2013	−4.7 (−22.3, 16.7)	2011–2017	−2.5 (−5.7, 0.8)
Trend2	2013–2019	1.7 (−1.8, 5.2)	2017–2019	−7.6 (−23.9, 12.3)
AAPC (95%CI)	2011–2019	0.1 (−3.9, 4.1)	2011–2019	−3.8 (−7.4, −0.1)^*^
**High**				
Trend1	2011–2013	−12.3 (−31.5, 12.3)	2011–2013	30.9 (−5.6, 50.5)
Trend2	2013–2019	9.8 (5.3, 14.5)^*^	2013–2019	0.7 (−8.5, 10.8)
AAPC (95%CI)	2011–2019	3.8 (−1.1, 9.0)	2011–2019	7.5 (−3.9, 20.3)
**Occupation**				
**Professional services**				
Trend1	2011–2013	−8.4 (−31.0, 21.7)	2011–2013	30.8 (−7.6, 84.9)
Trend2	2013–2019	7.9 (2.9, 13.2)^*^	2013–2019	−3.2 (−8.7, 2.7)
AAPC (95%CI)	2011–2019	3.6 (−2.1, 9.6)	2011–2019	4.4 (−2.5, 11.8)
**Housewives**				
Trend1	2011–2013	−6.2 (−25.5, 18.1)	2011–2013	−9.2 (−24.7, 9.4)
Trend2	2013–2019	−1.3 (−5.0, 2.7)	2013–2019	1.4 (−1.8, 4.6)
AAPC (95%CI)	2011–2019	−2.5 (−6.8, 2.0)	2011–2019	−1.4 (−5.0, 2.3)
**Manual workers**				
Trend1	2011–2014	−18.3 (−37.1, 6.0)	2011–2015	−18.2 (−47.8, 28.3)
Trend2	2014–2019	14.2 (1.6, 28.3)^*^	2015–2019	6.6 (−32.0, 67.2)
AAPC (95%CI)	2011–2019	0.7 (−7.6, 9.7)	2011–2019	−6.6 (−25.4, 16.9)

APC, annual percentage change; AAPC, average annual percent change; CI, confidence interval, ^*^p < 0.05.

## Discussion

In the current study, we found that among singleton pregnancies, women with C-sections had a significantly higher incidence of several adverse maternal–neonatal outcomes compared with normal women. However, women with C-sections showed significantly lower incidences of LBW, perinatal mortality, LPI, and low Apgar scores compared with normal women in both singleton and twin pregnancies. Among singleton pregnancies, AMA, previous history of C-section, HDP, abnormal placentation, GDM, oligohydramnios, polyhydramnios, fetal breech presentation, macrosomia, and fetal distress were prominent factors associated with an increased odds of C-section. Among women with twin gestations, HDP significantly increased the risk of C-section. A previous C-section was associated with a higher likelihood of GDM in the subsequent pregnancy of both women with singleton and twin gestations. The secular trend of C-section among singleton and twin pregnancies was not significantly increased between 2011 and 2019. However, the trend of C-sections was significantly increased among singleton women with high education levels and professional services between 2013 and 2019.

### Baseline distribution of adverse pregnancy outcomes by mode of delivery and factors of C-section

We observed a high rate of cesarean delivery, affecting 60.7% of women with singleton and 80.7% of those with twin pregnancies. These rates are higher than those reported in previous Chinese studies. This is likely because our study was conducted in a tertiary hospital, which admits a higher proportion of high-risk pregnancies. Therefore, these results should not be generalized to the broader national population. According to a WHO survey, China had 46.2% C-sections during 2007-2008 (2). The C-section rate was 39.3% at the national level in 2008 (4). Reported C-section rates in Eastern China range from 36.1% in Zhejiang Province (2012-2021) (32) to 49.7% in Jiangsu Province (2016–2019) (7). In 2018, the global C-section rate was 21.1%, with averages of 8.2% in the least developed, 24.2% in less developed, and 27.2% in more developed regions. The Dominican Republic (58.1%), Brazil (55.7%), and Cyprus (55.3%) lead globally, with Egypt and Turkey also exceeding 50%. Romania has the highest rate in Europe (46.9%). The five countries with the world's lowest C-section rates are all in Africa, led by Chad and Niger (both 1.4%). The lowest rates in other regions are also significantly low, such as Papua New Guinea (3.0%) in Oceania and Timor-Leste (3.5%) in Asia (33).

Moreover, women with C-sections had a significantly higher incidence of GDM, HDP, abnormal placentation, oligohydramnios, fetal breach presentation, preterm births, macrosomia, and fetal distress compared with normal women in singleton pregnancies. However, women with C-sections showed significantly lower incidences of LBW, perinatal mortality, LPI, and low Apgar scores compared with normal women in both singleton and twin pregnancies. The factors for C-section were AMA, previous history of C-section, HDP, abnormal placentation, GDM, oligohydramnios, polyhydramnios, fetal breech presentation, macrosomia, and fetal distress among singleton pregnancies, and HDP significantly increased the risk of C-section among twin pregnancies, which is supported by the previous reports (8, 9).

In a multicenter retrospective cohort study including 16 hospitals, Wang et al. found that parity and advanced maternal age were prominent factors associated with a higher risk of C-section in Chinese women (18). In another multicenter cross-sectional study consisting of 39 hospitals, the factors associated with a higher rate of C-section were cesarean delivery on maternal request (CDMR), cephalo-pelvic disproportion, fetal distress, macrosomia, breech presentation, and previous C-section (5). However, our study failed to consider CDMR as a factor in our analysis due to a lack of data. It has been observed that CDMR accounted for 28.4% of all C-section deliveries in mainland China (5). Some studies found that higher pre-gestational body mass index (BMI), maternal age >30 years, excess gestational weight gain (34), AMA, and a private hospital were prominent indicators for a higher rate of C-section in China (35).

In the US population, the leading four factors for higher C-section rates were dystocia, previous C-section, breech presentation, and fetal distress (36), and in China, the leading factors for C-section were CDMR, cephalo-pelvic disproportion, fetal distress, and previous C-section (5). Unlike these previous studies (5, 36), our findings showed that the five leading factors were previous C-sections, followed by fetal breech presentation, abnormal placentation, oligohydramnios, and macrosomia in our study. However, a previous C-section is a common factor for a higher risk of C-sections in the prior studies (5, 36) and in our findings. In a survey conducted in China, the main factor for C-section was changed from cephalo-pelvic disproportion in 1999 to a previous C-section in 2009 (37). Similarly, another study reported that previous C-section was a prominent factor for performing C-sections in China (38), and the rate of C-section attributable to previous C-sections increased by 190% in 3 years (38).

### Impact of previous C-section on the subsequent pregnancy outcomes

It is widely known that a previous C-section is a predominant risk factor associated with a higher risk of abnormal placentation, including placenta previa and placenta accreta. Abnormal placentation may be associated with fatal bleeding at delivery and causes maternal morbidity and mortality (15, 39). However, in our study, a previous C-section does not significantly increase the risk of HDP and abnormal placentation in the subsequent pregnancy of both singleton and twin women. A study conducted by Jacob et al. (14) in Germany reported that a previous C-section is significantly associated with a higher risk of gestational hypertension, preeclampsia, and polyhydramnios in the following pregnancy compared with vaginal delivery.

We observed that a previous C-section was significantly associated with a higher likelihood of GDM in women with both singleton and twin gestations. This finding is consistent with prior studies, which have also reported an association between a prior C-section and GDM in subsequent singleton pregnancies (24, 40). However, the association between a previous C-section and GDM in subsequent twin pregnancies has not been addressed in prior research (24, 40). The observed association between a prior C-section and subsequent GDM is likely multifactorial. The findings suggest a non-causal relationship, potentially explained by confounding variables such as shared pre-existing risk factors, particularly maternal obesity and chronic insulin resistance, which predispose women to both a primary C-section and GDM in a future pregnancy (41–43). For example, maternal obesity increased the risk of GDM and C-section (43, 44), and a prior pregnancy complicated by GDM is at increased risk for subsequent GDM (41). A notable limitation of our study is the lack of data on maternal obesity, a known potential confounder of the association between previous C-section and GDM. Moreover, a previous C-section is significantly associated with a lower risk of preterm births and a prolonged pregnancy (14). We also observed that a previous C-section was associated with a lower risk of PROM, nuchal cord, preterm birth, LBW, perinatal mortality, and low Apgar score among singleton pregnancies.

We showed that a previous C-section significantly increases the risk of C-sections by 3.4-fold in the following pregnancy among women with singleton gestation. Interestingly, a previous C-section is not significantly associated with a higher risk of C-sections in women with twin gestations in our study. It suggests that twin gestation may be a risk factor by itself for C-sections. In a Nigerian population, Iyoke et al. (15) found that women with a previous C-section were associated with a higher risk of C-section, placenta previa, labor dystocia, and intrapartum hemorrhage in the subsequent pregnancy compared with women who had a previous vaginal delivery. In a German population, a previous C-section was associated with a 36-fold higher risk of C-section in the subsequent pregnancy (14). Our findings and previous studies (14, 15) revealed that a previous C-section is associated with higher odds of C-section in the following pregnancy. Little is known about the actual impact of previous C-sections on subsequent C-sections (45, 46).

### Secular trend of C-section (2011–2019)

Between 2011 and 2019, the temporal trend of C-section was not significantly increased among both singleton and twin pregnancies in our study. However, the C-section rate increased from 3.4% to 39.3% at the national level in China between 1988 and 2008 (4). Moreover, the C-section rate increased from 2.0% in 1978 to 54.9% in 2011 (5, 6). On the other hand, C-section rate declined from 52.5% in 2012–2015 to 49.7% in 2016–2019 in Jiangsu province, China (7). We observed that the secular trend of C-section was significantly increased among singleton women with a high education level (annual percentage change (APC), 9.8%; 95%CI: 5.3, 14.5) and professional services (APC, 7.9%; 95%CI: 2.9, 13.2) between 2013 and 2019. The observed increase in C-sections between 2013 and 2019 may be partially attributable to China's universal two-child policy, enacted during this period. This policy likely increased the proportion of births among women of AMA, which rose by 68.8% following the policy change (from 12.5% in the one-child policy period to 21.1% in the universal two-child policy period). Given that AMA is an independent risk factor for C-section, this demographic shift contributed to the overall rising trend, as evidenced by a significant increase in the C-section rate in women with AMA between 2014 and 2019 (30).

Women's higher socioeconomic status has always been a significant factor in higher C-section rates. A meta-analysis conducted in sub-Saharan Africa showed that maternal education and wealth index were significantly associated with higher utilization of C-section delivery (47). In the Indonesian population, women with higher education levels had a 3.2-fold higher C-section rate compared with those with lower education levels (48). In developing countries, women with a higher socioeconomic status and better access to antenatal care services are the most likely to undergo a C-section (49). Well-educated women feel that a C-section is safer, causes less pain, is less disruptive to their job and leisure time, and is more socially prestigious than a vaginal birth (49). According to the International Federation of Gynecology and Obstetrics (FIGO), non-medically indicated C-sections are not within the parameters of excellent professional practice. Medically indicated C-sections should be undertaken to improve the wellbeing of mothers and babies and pregnancy outcomes (50). Although our study lacked data on non-medically indicated C-sections, the findings suggest that future public health initiatives could consider providing education on the potential consequences of a primary C-section for subsequent pregnancies. This may be particularly relevant for highly educated women and those in professional services, who demonstrated a rising trend in C-section rates in this study.

Our study had several limitations. First, we conducted a mono-center study in a tertiary care hospital, which could be a selection bias in the present study. Second, our study is missing several factors, including maternal smoking, pre-pregnancy BMI, gestational weight gain, and assisted reproductive technology, which may bias our findings. Third, we failed to categorize C-sections into medically indicated, non-medically indicated, and cesarean deliveries on maternal request in our study. Fourth, due to the small sample size and single-center study, the results cannot be generalized to the whole population.

## Conclusion

In summary, several maternal–neonatal factors in singleton pregnancy and HDP in twin pregnancy are associated with a higher likelihood of C-section. A history of prior C-section was associated with a higher likelihood of GDM in subsequent pregnancies for both singleton and twin gestations. Furthermore, a previous C-section was a strong predictor of repeated C-sections in subsequent singleton pregnancies. The secular trend of C-section rate was significantly increased among singleton women with high education levels and professional services between 2013 and 2019. Collectively, these findings highlight a potential cycle where a primary C-section is linked to adverse outcomes in subsequent pregnancies, which may in turn influence delivery decisions. Therefore, the observed associations warrant further investigation into the decision-making processes behind C-sections, especially among higher-educated women. Future research should also explore whether targeted health education about the potential consequences of a primary C-section could be a valuable component of strategies aimed at optimizing delivery outcomes in the Chinese population.

## Data Availability

The original contributions presented in the study are included in the article/Supplementary material, further inquiries can be directed to the corresponding authors.
